# Adapt or perish: SARS-CoV-2 antibody escape variants defined by deletions in the Spike N-terminal Domain

**DOI:** 10.1038/s41392-021-00601-8

**Published:** 2021-04-24

**Authors:** Marta Ribes, Carlos Chaccour, Gemma Moncunill

**Affiliations:** 1 ISGlobal, Hospital Clínic - Universitat de Barcelona, Barcelona, Spain; 2 Ifakara Health Institute, Ifakara, United Republic of Tanzania; 3 Facultad de Medicina, Universidad de Navarra, Pamplona, Spain

**Keywords:** Infectious diseases, Genetics

In a recent report in *Science*, McCarthy et al. promptly observed a pattern of recurrent deletions in four discrete regions of the N-terminal Domain (NTD) of the Spike protein, which provide resistance to antibody neutralization, suggesting convergent evolution due to selective pressure and antigenic drift.^1^

Evolution is nature’s inexorable imperative. A year into the COVID-19 pandemic, the rollout of vaccines opens a window of hope to end transmission in many countries, yet, recent concerns have arisen due to the SARS-CoV-2’s *gambits* to escape the immune system and to sustain its spread.^2^ Given that coronaviruses encode an exoribonuclease that provides a proofreading function during the replication, the appearance of variants was at first expected to be marginal. McCarthy’s work anticipated the events, as these deletions were found to be present in some of the lineages causing now a global concern.

McCarthy et al. founded their research on the observation of an immunocompromised cancer patient who succumbed to the COVID-19 infection 74 days after diagnosis. They sequenced the S genes from samples obtained 72 days after diagnosis and identified two variants with deletions in the NTD. Avanzato et al. had reported a similar case,^3^ illustrating SARS-CoV-2 adaptation to selective pressure within a single human being. These findings prompted them to look for similar cases in the GISAID database. Remarkably, they found eight patients with samples from late time points having viruses with deletions within the S gene that were not present at early time points. Tellingly, these viruses could be distinguished from one another by nucleotide differences and appertained to monophyletic clades, suggesting that within each individual, the repeated pattern of deletions was the result of adaptation and not of onward transmission.

**Fig. 1:**
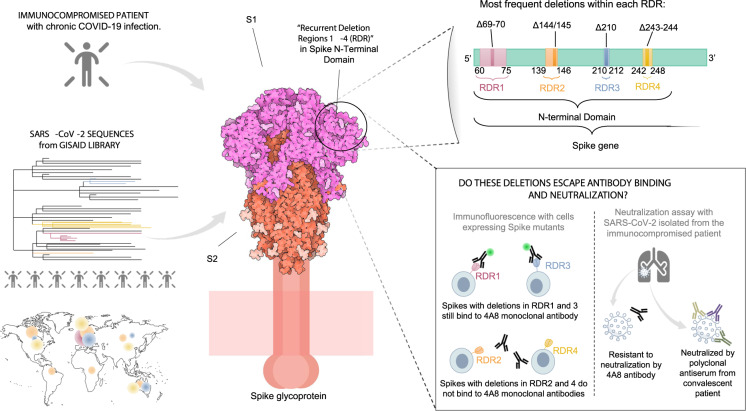
A pattern of recurrent deletions in the NTD of the Spike was observed by McCarthy et al. RDRs were identified in an immunocompromised patient, in late but not baseline samples of eight COVID-19 patients and in 1108 sequences originating in diverse geographic sites. Center: representation of SARS-CoV-2 spike by David S. Goodsell and the Research Collaboratory for Structural Bioinformatics Protein Data Bank. Top right: the location of the RDRs is represented and delimited by the numbered amino acid. Bottom right: indirect immunofluorescence showed that the RDR2 and RDR4 deletions in S expressed on the surface of Vero E6 cells abrogated the binding of the 4A8 monoclonal antibody. Non-plaque-purified viral population from the immunocompromised patient showed resistance to neutralization by 4A8, but was neutralized by polyclonal antisera from convalescent patients

McCarthy et al. analyzed a total of 146,795 sequences in GISAID and identified 1108 with deletions in the S gene with 90% of them occupying four discrete sites within the NTD, which they termed “recurrent deletion regions 1–4” (RDRs). Although with a degree of variance, RDR1 typically harbored the Δ69–70 deletion, RDR2 the Δ144/145, RDR3 the Δ220, and RDR4 the Δ243–244 deletion (Fig. 1). In these RDRs, >97% of the deletions preserved the reading frame, which is largely over the probabilistic rate at which in-frame mutations occur. Besides, the phylogenetic analysis showed independent distinct branches from diverse geographic origins that were followed by onward community transmission, pointing again toward a convergent outcome to evade a common selective pressure.

RDRs 1 and 3 and RDRs 2 and 4 occupy different surfaces on the S glycoprotein that map antibody epitope regions (Fig. 1). To assess whether these deletions confer an advantage vis-à-vis the host’s immune system, McCarthy et al. created plasmids with S genes harboring the RDR deletions and transfected them into cells. With indirect immunofluorescence, they observed that the 48A- neutralizing monoclonal antibody did not bind to the S protein when carrying the deletions: Δ69–70 + Δ144/145 (RDR1 + 2), Δ141–145 or Δ144/145 or Δ146 in RDR2, and Δ243–244 in RDR4, while Δ210 in RDR3 and Δ69/70 alone in RDR1 did not abolish the binding. Overcoming the limitation of engineering viruses with the desired mutations, McCarthy’s team isolated the virus from the original cancer immunocompromised patient. The virus also resisted neutralization by 4A8 monoclonal antibody, but was, however, neutralized by polyclonal antiserum from other convalescent patients, a finding that is supported by another study.^4^ This suggests that deletions in the NTD of the S gene do not suffice in front of a battalion of neutralizing antibodies targeting different S epitopes.

The deletions identified by McCarthy et al. are present in variants of concern that have undergone rapid expansion. The Δ69–70 was present in the Mink Cluster, the Δ69–70 and Δ144/145 are found in the B.1.1.7 lineage, and the Δ242–244 in the B.1.351 lineage (first identified in South Africa). These lineages are typically defined by substitutions in the receptor-binding domain (RBD), the N501Y in the B.1.1.7 and B.1.351 lineages, and the E484K in the B.1.351. However, these are usually accompanied by other substitutions and deletions. A recent study observed that, far from being a mere companion, the Δ69–70 was key for the increased infectivity of the B.1.1.7 lineage: when reversed and other mutations like the N501Y maintained, the pseudotyped viruses lost substantial infectivity.^4^

Deletions in the NTD have been observed by several groups in immunosuppressed as well as in patients repeatedly treated with convalescent plasma, which would suggest an improvement in fitness by evading the host’s immune response. However, in McCarthy’s work, a virus carrying the deletions was still neutralized by polyclonal antisera. Another study also showed that viruses with Δ69/70 deletion, E484K and N501Y substitutions, were still neutralized by sera from BNT162b2 (Pfizer-BioNTech) vaccinated individuals.^5^

Mutations will still appear as long as the virus continues to replicate within hosts, as a matter of mere probabilities. So far, selective pressures toward the virus might have given rise to variants with a fitness advantage based on infection, replication, or transmission efficiency, although potential evasion from antibodies—like the one observed in McCarthy’s paper—might result as a by-product or due to immune pressure within an individual during chronic COVID-19. As the population becomes less naive to the virus through natural infection or vaccination, the selective pressure to escape acquired immune responses will be higher and will force the virus to find a way. Variants with antigenic drift would introduce antigenic novelty and enable reinfection, as happens with influenza A, whose hemagglutinin protein requires a very small number of mutations to evade pre-existing immunity.

H.G. Wells famously wrote “Adapt or perish, now as ever, is nature’s inexorable imperative”. This quote very elegantly summarizes evolution itself. In the midst of the COVID-19 pandemic, the virus never ceases to adapt, but neither do we. Thus, the best way forward to address variants of concern is to intensely monitor their emergence to be able to act proactively. Even if identification is limited by the lack of broadly established sequencing systems, the appearance of immune escape variants would probably be self-explanatory at the clinical level with vaccinated individuals recontracting the disease. In addition, one perk of mRNA-based vaccines is that they can rapidly be tweaked, or new sequences added for booster doses. Additional reasons for optimism are (a) T-cell responses may not be affected by the deletions and mutations of new variants as they recognize multiple antigens and epitopes conserved in the variants of concern and may provide protection beyond neutralizing antibodies, (b) even if vaccines fail to protect against infection or mild/moderate disease, they may still be effective against severe cases, and (c) reinfection with new variants may not lead to severe COVID-19. In the long run, although SARS-CoV-2 keeps evolving and adapting, so evolves our understanding of the virus and our tools to reduce its impact. Inexorably, the adapted virus must perish. We shall prevail.
